# ﻿Review of the leafhopper tribe Deltocephalini Dallas, 1870 (Hemiptera, Cicadellidae, Deltocephalinae) in Pakistan with description of a new species of *Paramesodes*

**DOI:** 10.3897/zookeys.1186.110266

**Published:** 2023-12-13

**Authors:** Hassan Naveed, Bismillah Shah, Kamran Sohail, Yalin Zhang, Keping Chen

**Affiliations:** 1 School of Food and Biological Engineering, Jiangsu University, Zhenjiang 212013, China; 2 School of Life Sciences, Jiangsu University, Zhenjiang 212013, China; 3 Department of Forestry Protection, School of Forestry and Biotechnology, Zhejiang A&F University, 666 Wusu Street, Linan, Hangzhou, Zhejiang 311300, China; 4 Department of Entomology, The University of Agriculture, Peshawar 25100, Pakistan; 5 Key Laboratory of Plant Protection Resources and Pest Management of the Ministry of Education, Entomological Museum, Northwest A&F University, Yangling, Shaanxi Province 712100, China

**Keywords:** Auchenorrhyncha, distribution, key, morphology

## Abstract

A review of the leafhopper tribe Deltocephalini in Pakistan is provided, including comprehensive illustrated keys to all genera and species, along with their diagnosis and distribution. A new species of *Paramesodes* is described, *P.dirensis***sp. nov.**, which was discovered in the Upper Dir District of Khyber Pakhtunkhwa, Pakistan. A detailed description of this new species is provided together with photographs for visual reference. This tribe now has three genera and 13 species from Pakistan. The genus *Deltocephalus* Burmeister includes two species, and *Maiestas* Distant has nine species; *Paramesodes* Ishihara is now represented with two species.

## ﻿Introduction

Leafhoppers of the tribe Deltocephalini can be identified by their small to moderate size, tapering or parallel-sided clypellus, narrow lorum, linear connective with closely appressed anterior arms, connective fused to the aedeagus, and dorsal sculpturing of the first valvula imbricate. This tribe includes 74 genera and over 600 species distributed worldwide (Duan et al. 2020; Zhang et al. 2023). Until now, 12 species of Deltocephalini have been known from Pakistan (Naveed et al. 2021; Shah et al. 2021).

The genus *Paramesodes* Ishihara, comprising 16 Old World species, is poorly represented in Pakistan, with only a single previous species, *P.lineaticollis* (Distant), known. Wilson (1983) revised the genus and recognized one species from Europe and the Middle East, one species from Africa, and nine species from Asia. Five other species were subsequently included: *P.montanus* Rao, (1989) from India, *P.wilsoni* Rao & Ramakrishnan, (1990) from India, *P.iraniensis* Webb & Viraktamath, (2009) from Iran, and *P.menghaiensis* Li, Dai & Xing, (2011) and *P.cangshanae* Duan & Zhang, (2012) from China. *Paramesodes* is externally similar to *Paramesus* Fieber, *Exitianus* Ball, and *Ctenurellina* McKamey but they can be differentiated by having the forewing with the inner anteapical cell open basally (closed in *Paramesus* and *Ctenurella*), and the veins are always pale or white (usually brown in *Exitianus*). In the male genitalia the connective and aedeagus are fused (free in *Paramesus* and *Exitianus*), and the pygofer side has a dense covering of long, stout, pale macrosetae (2–6 dark or black macrosetae along apical margin in *Exitianus*) and the pygofer side has a large process (lower edge of pygofer pectinate in *Ctenurella*). This, together with the aedeagus, is the most important character for species identification (Duan and Zhang 2012).

In this paper, we provide a list of leafhoppers of the tribe Deltocephalini from Pakistan, illustrated keys to genera and species with their diagnostic characters, and a description and illustration of a new species, *P.dirensis* sp. nov.

## ﻿Material and methods

All specimens were examined with a Leica ZOOM2000 stereomicroscope. Drawings were made using an Olympus drawing tube. Photos were taken by a ZEISS SteREO Discovery.V20 stereomicroscope equipped with a ZEISS AxiocamICc 5 camera that also provided measurements. Adobe Photoshop CS was used to compile photographs. Specimens from Pakistan are deposited in the various collections as indicated in the published records. Type specimens of the new species, examined and figured for this study, are deposited in the Entomological Museum, Northwest A&F University, Yangling, Shaanxi, China.

## ﻿Taxonomy

**Family Cicadellidae Latreille, 1802**

**Subfamily Deltocephalinae Dallas, 1870**

**Tribe Deltocephalini Dallas, 1870**

### ﻿Key to genera of Deltocephalini from Pakistan

**Table:** 

1	Crown with broad black transverse submarginal band between eyes (Figs 1, 9); pygofer side with large process (Figs 3, 10)	***Paramesodes***
–	Crown without black transverse band between eyes (Figs 12, 15, 18, 19); pygofer side without process	**2**
2	Aedeagal shaft short, robust, strongly curved dorsally, gonopore apical (Figs 13, 16)	***Deltocephalus***
–	Aedeagal shaft long, slightly curved dorsally, with gonopore indistinct (Figs 20–25, 27, 29)	***Maiestas***

**Genus *Deltocephalus* Burmeister**

### ﻿Key to species of *Deltocephalus* from Pakistan (male)

**Table:** 

1	Crown with six brown spots on anterior margin (Fig. 12); aedeagal shaft with shallow apical notch (Fig. 13)	***D.vulgaris***
–	Crown with single brown spot on anterior margin adjacent to eyes (Fig. 15); aedeagal shaft without apical notch (Fig. 16)	***D.infirmus***

#### ﻿ Deltocephalus vulgaris

Taxon classificationAnimaliaHemipteraCicadellidae

Dash & Viraktamath

03ABBA5E-7CB4-5FB4-8A20-19CD71DD9DCE

Figs 12–14

Deltocephalus (Deltocephalus) vulgaris Dash & Viraktamath, 1998: 4, figs 1–11 (India); Zhang and Duan 2011: 3, fig. 3A–H (China); Naveed et al. 2019: 285, figs 1A, B, 3A–D (Pakistan).

##### Diagnosis.

This species can easily be identified by the color pattern and the aedeagus with a shallow apical notch.

##### Distribution.

China, India, Pakistan.

#### ﻿ Deltocephalus infirmus

Taxon classificationAnimaliaHemipteraCicadellidae

Melichar

AA36E19E-AC1B-5093-94B5-4D3EF381BDC1

Figs 15–17

Deltocephalus infirmus Melichar, 1903: 203, pl. 5 fig. 11 (Sri Lanka); Jassargusinfirmus: Ishihara 1961: 244, figs 53–58 (misidentification); Deltocephalusinfirmus: Webb and Viraktamath 2009: 13, fig. 10; Naveed et al. 2019: 285, figs 1C, 3D–G (Pakistan).

##### Diagnosis.

A similar species to *D.vulgaris* but differs by having the crown with only a single brown spot and the aedeagal shaft without an apical notch.

##### Distribution.

India, Pakistan, Sri Lanka, Thailand.

**Genus *Maiestas* Distant**

### ﻿Key to species of *Maiestas* from Pakistan (males)

**Table:** 

1	Overall color dark brown; forewing with sub basal and subapical irregular white transverse band (Fig. 18)	***M.albomaculata***
–	Color not as above (Fig. 19)	**2**
2	Crown, face, and thorax with black patches (Fig. 19)	***M.maculata***
–	Crown, face, and thorax without black patches	**3**
3	Forewing with extra cross-veins, at least in clavus	**4**
–	Forewing without extra cross-veins	**5**
4	Aedeagus with a large subapical ventral process (Fig. 20)	***M.indica***
–	Aedeagus with a short apical ventral process (Figs 21, 22)	***M.pruthii***
5	Aedeagus with pair of short lateral processes (Fig. 23)	***M.trispinosa***
–	Aedeagus without lateral processes	**6**
6	Aedeagus in lateral view similar in width in distal half (Fig. 24)	***M.subviridis***
–	Aedeagus in lateral view evenly tapered from base to apex	**7**
7	Style apophysis broadest sub basally (Fig. 26); aedeagal shaft in lateral view not sinuate (Fig. 25)	***M.tareni***
–	Style apophysis broadest at base (Fig. 28); aedeagal shaft in lateral view slightly sinuate (Fig. 27)	***M.sinuata***

#### ﻿ Maiestas albomaculata

Taxon classificationAnimaliaHemipteraCicadellidae

(Dash & Viraktamath)

F913A654-EB9C-5FCF-A6BB-6705D95C63C1

Fig. 18

Deltocephalus (Recilia) albomaculatus Dash & Viraktamath, 1998: 12, figs 29–34 (India); Maiestasalbomaculata: Webb and Viraktamath 2009: 21; Maiestasalbomaculata: Naveed et al. 2019: 287, figs 1E–I, 3H, I (Pakistan); Shah et al. 2021: 403, fig. 1A–D (Pakistan).

##### Diagnosis.

This species differs from other species of *Maiestas* in color and male genitalia, including the dorsolateral, laminate serrations of the aedeagal shaft.

##### Distribution.

Pakistan, India.

#### ﻿ Maiestas indica

Taxon classificationAnimaliaHemipteraCicadellidae

(Singh-Pruthi)

24DD79D6-266A-5A56-9408-D64DBB8A5EB5

Fig. 20

Allophleps indica Singh-Pruthi, 1936: 120, fig. 132; pl. 9 fig. 3 (Pakistan); Allophlepsdelhiensis Rao & Ramakrishnan, 1990: 111, figs 1–9 (India), synonymized by Dash and Viraktamath 1998: 35; Deltocephalus (Recilia) indicus: Dash and Viraktamath 1998: 35–36, fig. 305 (India); Maiestasindica: Webb and Viraktamath 2009: 21; Naveed et al. 2019: 287; Shah et al. 2021: 403, fig. 1E (Pakistan).

##### Diagnosis.

This species can be identified by the aedeagus, which has a distinctive, large, subapical ventral process, the forewings which have accessory cross-veins, and the shorter head.

##### Distribution.

Pakistan, India.

#### ﻿ Maiestas maculata

Taxon classificationAnimaliaHemipteraCicadellidae

(Singh-Pruthi)

F7C0F730-7BA9-5A13-9168-98391CD4F16E

Figs 19 , 29

Cicadula maculata Singh-Pruthi, 1930: 58–59, figs 80, 81, pl. 5 fig. 2 (India); Thamnotettixprabha Singh-Pruthi, 1930: 62, figs 85, 86, pl. 5 figs 6, 6a (India), synonymized by Webb and Viraktamath 2009: 41; Reciliaprabha: Ghauri 1980: 166–169, figs 1, 3–11; Deltocephalus (Recilia) maculata: Dash and Viraktamath 1998: 32, figs 260–269 (India); Maiestasmaculata: Webb and Viraktamath 2009: 22; Maiestasmaculata: Zhang and Duan 2011: 37–39, figs 33–35, pl. 4 fig. E, pl. 5 fig. P, pl. 6 fig. P (China); Shah et al. 2021: 404, fig. 2A–I (Pakistan).

##### Diagnosis.

This species can be distinguished from other *Maiestas* species by its habitus, which has variable black patches on the head and thorax, and the shape of its aedeagus and style.

##### Distribution.

China, India, Pakistan.

#### ﻿ Maiestas pruthii

Taxon classificationAnimaliaHemipteraCicadellidae

(Metcalf)

1A88C146-2B57-5AC2-9D08-26A8D3D575AC

Figs 21 , 22

Deltocephlaus notatus Singh-Pruthi, 1936: 128–129, fig. 139, pl. 9 fig. 10 (Pakistan) (primary homonym: Deltocephalusnotatus Melichar, 1896); Deltocephaluspruthii Metcalf, 1967: 1173 (nom. nov. pro D.notatus Singh-Pruthi, 1936); Deltocephalus (Recilia) pruthii: Dash and Viraktamath 1998: 22, 23, figs 150–158 (India); Maiestaspruthii: Webb and Viraktamath 2009: 20, new combination; Khatri and Webb 2010: 11, pl. 2a fig. 13, misidentification; Naveed et al. 2019: 286, fig. 2A–C (incorrectly cited as M.subviridis; H. Naveed pers. comm.), Fig. 3i, misidentification; Shah et al. 2021: 406, fig. 4F–L (Pakistan).

##### Diagnosis.

This species has a relatively long, acute head, with a pair of inverted U-shaped markings basally, and forewings with extra cross veins. The identity of this species is based on the figures of Dash and Viraktamath (1998).

##### Distribution.

India, Pakistan.

#### ﻿ Maiestas setosa

Taxon classificationAnimaliaHemipteraCicadellidae

(Ahmed, Murtaza & Malik)

7F8F210F-3844-58B0-9C87-9322E95184B8

Recilia setosa Ahmed et al., 1988: 412, fig. 2 (Pakistan); Maiestassetosa: Webb and Viraktamath 2009: 20; Naveed et al. 2019: 287; Shah et al. 2021: 406 (Pakistan).

##### Diagnosis.

The identity of this species remains uncertain due to the limitations of the original description and the accompanying figures. Additionally, the type series from Karachi, which was indicated in the original account as deposited in the Zoological Museum of the University of Karachi (Ahmed et al. 1988), is unavailable (Khatri and Webb 2010: 11). Until the type material can be studied, pinpointing the precise classification of this species will be challenging.

##### Distribution.

Pakistan.

#### ﻿ Maiestas sinuata

Taxon classificationAnimaliaHemipteraCicadellidae

Shah & Duan

A67B45D0-BAFE-5AB2-BFF9-9912013D5548

Figs 27 , 28

Maiestas sinuata Shah & Duan in Shah et al., 2021: 406, fig. 3A–H (Pakistan).

##### Diagnosis.

This species differs in appearance of its habitus, presence of fine, apical setae on the subgenital plate, the style having a thicker apical process than other species, and the aedeagus lacking a ventrobasal “heel”.

##### Distribution.

Pakistan.

#### ﻿ Maiestas subviridis

Taxon classificationAnimaliaHemipteraCicadellidae

(Metcalf)

50D9A92C-7DF9-57FD-A796-22DEBE569D56

Fig. 24

Stirellus subviridis Metcalf, 1946: 125; Deltocephalus (Recilia) subviridis: Dash and Viraktamath 1998: 24, figs 166–172 (India); Maiestassubviridis: Webb and Viraktamath 2009: 19, fig. 40; Maiestassubviridis: Khatri and Webb 2010: 11, pl. 2b, c, fig. 12 (Pakistan); Zhang and Duan 2011: 19, fig. 17, pl. 2 fig. H (China); Naveed et al. 2019: 287; Shah et al. 2021: 408, fig. 4A–E (Pakistan).

##### Diagnosis.

This species can be distinguished by the rounded apex of the aedeagus which bears a very short apical spine.

##### Distribution.

China, India, Pakistan, Pacific.

#### ﻿ Maiestas tareni

Taxon classificationAnimaliaHemipteraCicadellidae

(Dash & Viraktamath)

7925FAB6-B0F7-540B-846E-EA98B2B43A96

Figs 25 , 26

Deltocephalus (Recilia) tareni Dash & Viraktamath, 1995: 74–76, figs 1–15; Dash and Viraktamath 1998: 16, figs 78–84 (India); Maiestastareni: Webb and Viraktamath 2009: 22; Khatri and Webb 2010: 11, pl. 2d, fig. 11 (Pakistan); Zhang and Duan 2011: 20 (China); Naveed et al. 2019: 290, figs 2G–I, 3N, O; Shah et al. 2021: 408, fig. 5A–Z (Pakistan).

##### Diagnosis.

This species can be identified by its relatively straight and stout style, apophysis with a serrated inner margin, and the aedeagus in lateral view evenly tapered from base to apex and relatively straight.

##### Distribution.

China, India, Pakistan.

#### ﻿ Maiestas trispinosa

Taxon classificationAnimaliaHemipteraCicadellidae

(Dash & Viraktamath)

4B351AAC-875C-5F94-96F8-DE09ECA87068

Fig. 23

Deltocephalus (Recilia) trispinosus Dash & Viraktamath, 1998: 35, figs 296–304 (India); Maiestastrispinosa: Webb and Viraktamath 2009: 38; MaiestastrispinosaShah et al., 2021: 408, fig. 6A–I (Pakistan).

##### Diagnosis.

This species can easily be distinguished from the others by the lateral, spine-like processes of the aedeagus.

##### Distribution.

India, Pakistan.

#### ﻿Genus Parasmesodes

Taxon classificationAnimaliaHemipteraCicadellidae

Ishihara

B05D2E66-D20C-561A-9DE1-B4268326F378

Paramesodes Ishihara, 1953: 45. Type species: Athysanusalbinervosus Matsumura, 1902.

##### Distribution.

Palearctic, Oriental, and Ethiopian regions.

##### Remarks.

Previously, only 1 species was recorded from Pakistan. This study adds one more new species to the genus, bringing the total to two for the country.

### ﻿Key to species of *Paramesodes* from Pakistan (males)

**Table:** 

1	Pale yellowish species (Fig. 1); pygofer broadly rounded posteriorly, with process in lateral view straight distally, apex directed posteriorly (Fig. 3); aedeagal shaft recurved distally in lateral view (Fig. 7); constricted subapically in ventral view (Fig. 6)	***P.dirensis* sp. nov.**
–	Dark yellowish species (Fig. 9); pygofer oval posteriorly, with process in lateral view directed dorsally distally (Fig. 10); aedeagal shaft evenly curved dorsally in lateral view (Fig. 10), not constricted preapically in ventral view (Fig. 11)	***P.lineaticollis***

#### ﻿ Paramesodes dirensis sp. nov.

Taxon classificationAnimaliaHemipteraCicadellidae

2C6398CA-B87E-5181-81A4-564F8E236F76

https://zoobank.org/158C20E7-3752-4E06-A4CE-AED5721D0A7D

Figs 1–8

##### Description.

**Figures 1–8.**
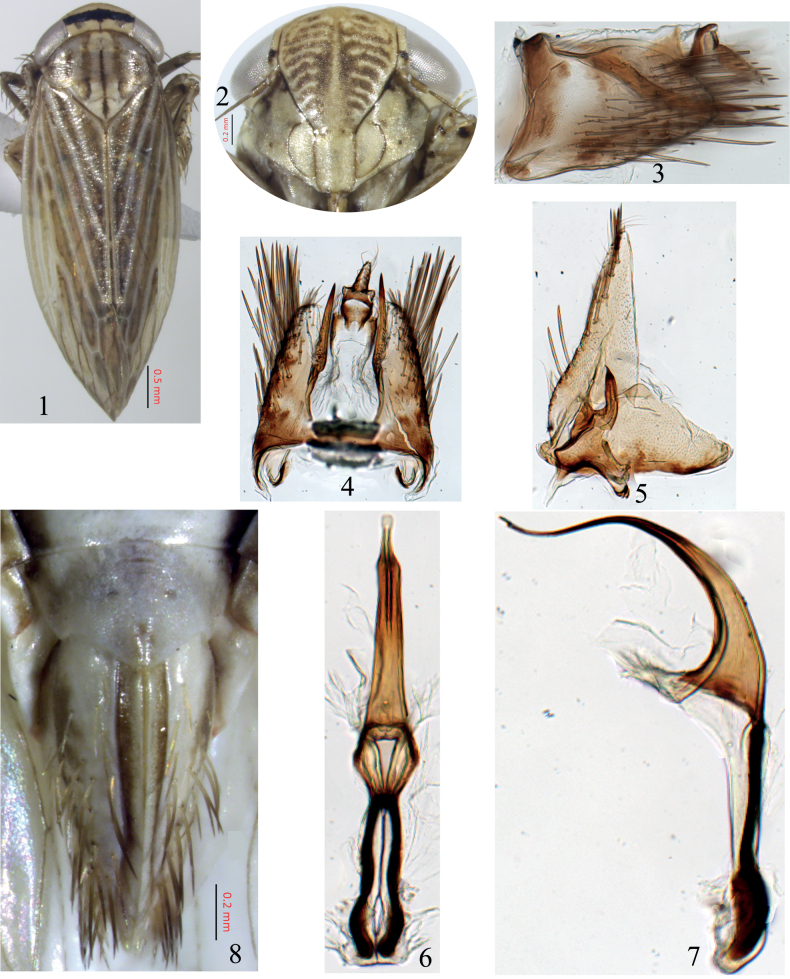
*Paramesodesdirensis* sp. nov. **1** habitus, dorsal view **2** face **3** male pygofer, lateral view **4** male pygofer, dorsal view **5** subgenital plate, valve and style, dorsal view **6** connective and aedeagus, dorsal view **7** connective and aedeagus, lateral view **8** female abdominal tip and 7^th^ sternum, dorsal view.

***Length***: male 5.2–6.1 mm, female 6.1–6.4 mm. ***Coloration***: pale, with brown markings (Fig. 1). Crown with broad, black, transverse submarginal band between eyes (Fig. 1). Face pale yellow, with brown, transverse striations on clypeus (Fig. 2). Pronotum with medial dark brown longitudinal marking as well as three brown longitudinal markings on each side (Fig. 1). Scutellum with median, longitudinal, dark brown markings and pale brown lateral markings (Fig. 1). Forewings with variable brown markings; veins prominent and white (Fig. 1). Legs pale, with brown markings.

***Male genitalia***: pygofer lobe broad basally, narrowing apically, forming a rounded-oval apex, with long, yellowish-brown spines extending beyond apical margin, a large process arising near medial dorsal margin and straight apically, surpassing pygofer lobe, without any bend (Figs 3, 4). Subgenital plates triangulate; macrosetae uniseriate laterally (Fig. 5). Valve triangular (Fig. 5). Style as in Figure 5. Connective fused to aedeagus, with arms closely appressed distally (Figs 6, 7). Aedeagus tubular, tapering apically, recurved in lateral view, constricted preapically in ventral view; gonopore apical (Figs 6, 7).

**Female.** Same in appearance as male. Seventh sternum with lateral margins not extended, posterior margin with median projection, rounded (Fig. 8).

##### Materials examined.

***Holotype*** ♂, Pakistan: Khyber Pakhtunkhwa: Upper Dir, 35°9'55.89"N, 72°2'48.54"E, 1840 m, 24.07.2019, Hassan Naveed leg., sweep net. ***Paratypes*** 8♂, 5♀, same data as holotype.

##### Etymology.

This species is named after type locality, the Upper Dir in Khyber Pakhtunkhwa.

##### Remarks.

*Paramesodesdirensis* sp. nov. is similar to its congeners in general appearance, but it differs from those species in the combination of male genitalia features, i.e., the pygofer is oval posteriorly with a relatively straight process distally and the aedeagal shaft is distally recurved in lateral view and constricted preapically in ventral view. In the Wilson’s (1983) key, the new species runs to couplet 7 along with *P.lineaticollis*.

#### ﻿ Paramesodes lineaticollis

Taxon classificationAnimaliaHemipteraCicadellidae

(Distant)

053D31F1-B00C-5C58-96A9-C0855E0A97B2

Figs 9–11

Paramesodes lineaticollis (Distant, 1908: 294, Paramesus) (India); Wilson 1983: 21, 22, figs 23–29.
Paramesodes ishurdii Mahmood & Meher, 1973: 135 (Pakistan), synonymized by Wilson 1983: 21.

##### Materials examined.

♂, Pakistan: Khyber Pakhtunkhwa: Shinkiari, 34°28'19.1064"N, 73°16'14.3004"E, 22.07.2018, Bismillah Shah leg., sweep net.

##### Distribution.

Bangladesh, China, India, Indonesia, Pakistan, Philippines, Taiwan, Turkey.

##### Diagnosis.

The male pygofer processes is distinct, directed ventrally or postero-ventrally over basal half, and turned abruptly posteriorly immediately distad of its midlength. Mahmood and Meher (1973) reported this species as *P.ishurdii* for the first time from Pakistan, but that species was later synonymized by Wilson (1983).

**Figures 9–11.**
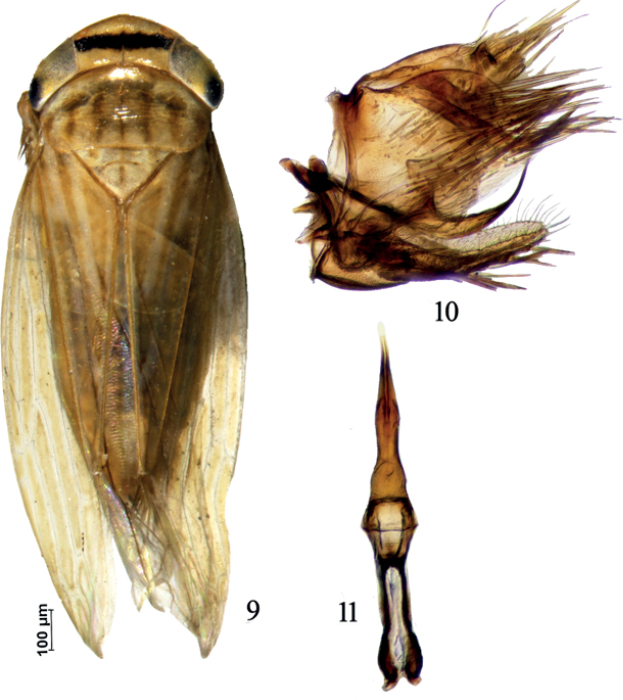
*Paramesodeslineaticollis***9** habitus, dorsal view **10** male pygofer, lateral view **11** connective and aedeagus, dorsal view.

**Figures 12–29.**
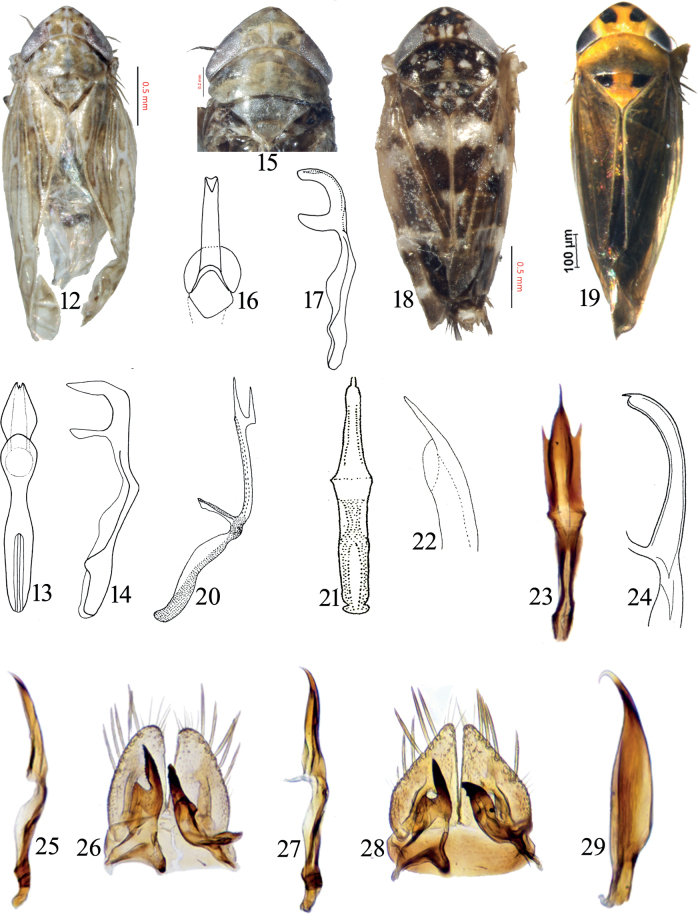
**12–14***Deltocephalusvulgaris* Dash & Viraktamath: **12** habitus, dorsal view **13** aedeagus and connective, dorsal view **14** aedeagus and connective, lateral view **15–17***D.infirmus* (Melichar): **15** habitus, dorsal view **16** aedeagus and connective, dorsal view **17** aedeagus and connective, lateral view **18***Maiestasalbomaculata* (Dash & Viraktamath) habitus, dorsal view **19***M.maculata* (Singh-Pruthi) habitus, dorsal view **20***M.indica* (Singh-Pruthi) aedeagus and connective, lateral view (after Dash & Viraktamath, 1998) **21, 22***M.pruthii* (Metcalf): **21** aedeagus and connective, dorsal view (after Dash & Viraktamath, 1998) **22** apex of aedeagus, lateral view **23***M.trispinosa* (Dash & Viraktamath) aedeagus and connective, dorsal view **24***M.subviridis* (Metcalf) aedeagus, lateral view (after Khatri & Webb, 2010) **25, 26***M.tareni* (Dash & Viraktamath): **25** aedeagus and connective, lateral view **26** subgenital plate, valve and styles, dorsal view **27, 28***M.sinuata* Shah & Duan: **27** aedeagus and connective, lateral view **28** subgenital plate, valve and styles, dorsal view **29***M.maculata* (Singh-Pruthi) aedeagus, lateral view.

**Figures 30, 31.**
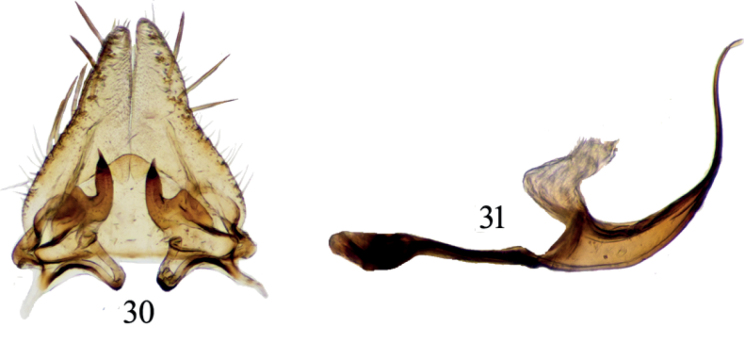
*Paramesodeslineaticollis***30** subgenital plates, valve and styles, dorsal view **31** aedeagus and connective, lateral view.

## Supplementary Material

XML Treatment for Deltocephalus vulgaris

XML Treatment for Deltocephalus infirmus

XML Treatment for Maiestas albomaculata

XML Treatment for Maiestas indica

XML Treatment for Maiestas maculata

XML Treatment for Maiestas pruthii

XML Treatment for Maiestas setosa

XML Treatment for Maiestas sinuata

XML Treatment for Maiestas subviridis

XML Treatment for Maiestas tareni

XML Treatment for Maiestas trispinosa

XML Treatment for Parasmesodes

XML Treatment for Paramesodes dirensis

XML Treatment for Paramesodes lineaticollis

## ﻿Additional information

### Author ORCIDs

Hassan Naveed https://orcid.org/0000-0002-9232-6299

Bismillah Shah https://orcid.org/0000-0002-8407-8627

Kamran Sohail https://orcid.org/0000-0003-1625-1130

Yalin Zhang https://orcid.org/0000-0002-1204-9181

Keping Chen https://orcid.org/0000-0001-5254-2299

### Data availability

All of the data that support the findings of this study are available in the main text.
